# Supplementary material for: Investigating Industrial Effluent Impact on Municipal Wastewater Treatment Plant in Vaal, South Africa
Source: Int J Environ Res Public Health. 2020 Feb 9;17(3):1096. doi: 10.3390/ijerph17031096 (PMC7037120; doi:10.3390/ijerph17031096)
Supplement: Supplementary file 1 [file ijerph-17-01096-s001.pdf]

Supplementary Material

Table S1. Variations of physico-chemical parameters (Mean ±SD) of industrial wastewater, Leeuwkuil WWTP inflow and final effluents across different seasons.

Parameters	Seasons	Sampling sites						
		**Industry 1**	**Industry 2**	**Industry 3**	**Industry 4**	**Industry 5**	**WWTP Inflow**	**WWTP effluent**
Temperature (ºC)	Summer	27.4 ±0.7	26.6 ±0.7	28.2 ±0.7	27.7 ±0.7	27.9 ±0.8	26.3 ±0.8	26.6 ±0.7
	Autumn	24.2 ±1.0	23.8 ±1.1	24.3 ±1.1	24.4 ±1.1	24.1 ±1.0	22.7 ±1.1	27.0 ±1.0
	Winter	16.4 ±0.8	15.7 ±0.8	16.2 ±0.8	15.8 ±0.7	16.0 ±0.9	16.6 ±0.7	16.7 ±0.8
	Spring	20.8 ±1.0	20.9 ±1.1	20.3 ±1.1	20.7 ±1.0	21.2 ±1.0	20.8 ±1.1	20.7 ±1.0
pH	Summer	6.9 ±1.1	8.7 ±1.1	9.2 ±1.1	10.8 ±1.0	10.5 ±1.0	9.0 ±1.0	7.7 ±1.1
	Autumn	6.8 ±0.9	8.2 ±1.0	10.2 ±1.0	7.7 ±1.0	10.3 ±1.0	7.9 ±0.9	8.2 ±0.9
	Winter	8.2 ±1.5	8.7 ±1.5	10.0 ±1.5	12.5 ±1.5	12.2 ±1.5	8.5 ±1.5	8.4 ±1.4
	Spring	7.0 ±1.8	6.4 ±1.8	9.4 ±1.8	12.5 ±1.7	11.5 ±1.9	7.8 ±1.7	7.7 ±1.8
BOD (mg/l)	Summer	5.8 ±1.8	5.6 ±1.8	4.2 ±1.8	5.4 ±1.6	6.5 ±1.8	4.9 ±1.8	3.3 ±1.8
	Autumn	5.7 ±2.3	7.0 ±2.3	6.6 ±2.2	5.1 ±2.2	6.7 ±2.3	3.7 ±2.2	3.1 ±2.2
	Winter	6.8 ±1.8	5.4 ±1.8	5.3 ±1.8	6.3 ±8.1	5.2 ±1.8	2.6 ±1.8	4.4 ±1.8
	Spring	5.6 ±1.8	5.9 ±1.8	5.3 ±1.8	6.9 ±1.8	5.3 ±1.8	2.1 ±1.8	4.2 ±1.8
DO (mg/l)	Summer	1.4 ±1.2	2.0 ±1.2	1.7 ±1.2	1.8 ±1.2	1.0 ±1.2	1.7 ±1.2	2.9 ±1.2
	Autumn	1.7 ±1.3	0.7 ±1.3	1.0 ±1.3	1.6 ±1.3	0.8 ±1.3	1.6 ±1.3	3.0 ±1.3
	Winter	1.2 ±1.59	2.1 ±1.6	2.2 ±1.7	2.1 ±1.6	1.1 ±1.6	1.3 ±1.6	2.2 ±1.7
	Spring	1.9 ±1.2	2.1 ±1.2	1.6 ±1.2	2.1 ±1.2	1.9 ±1.2	1.0 ±1.0	2.8 ±1.2
COD (mg/l)	Summer	228 ±189	277 ±206	909 ±589	621 ±423	275 ±21	456 ±368	3.4 ±2.3
	Autumn	320 ±280	1172 ±785	872 ±535	1128 ±783	670 ±460	360 ±29	3.0 ±2.7
	Winter	184 ±120	174 ±145	878 ±786	1209 ±934	200 ±16	468 ±32	136 ±99
	Spring	292 ±257	218 ±187	1493 ±936	714 ±635	95 ±7	452 ±35	0.3 ±0.1
TDS (mg/l)	Summer	1892 ±1145	2122 ±1256	22 ±15.7	2452 ±1632	3901 ±1987	328 ±304	265 ±204
	Autumn	2434 ±1436	1826 ±1189	19 ±14.9	3117 ±1826	2468 ±1357	354 ±323	253 ±199
	Winter	1845 ±1168	2223 ±1164	14 ±12.3	1417 ±1106	3636 ±1969	337 ±303	281 ±225
	Spring	1301 ±1127	1138 ±988	12 ±8.12	2335 ±1236	4611 ±2865	321 ±299	232 ±200
EC (µS/cm)	Summer	3603 ±2673	3782 ±2563	45 ±32.8	1954 ±1347	1425 ±1256	669 ±55	627 ±568
	Autumn	2743 ±1893	3594 ±2363	39 ±26.9	1797 ±1539	1109 ±986	710 ±68	505 ±475
	Winter	1845 ±1535	2223 ±1453	14 ±8.9	1417 ±1209	3636 ±2987	337 ±30	281 ±169
	Spring	2600 ±1895	2276 ±1468	25 ±18.9	4673 ±3673	9224 ±7869	642 ±54	464 ±370
Salinity (psu)	Summer	2.1 ±1.4	2.1 ±1.6	24.8 ±18.9	1.5 ±1.0	6.2 ±5.2	0.3 ±0.2	0.26 ±0.20
	Autumn	6.8 ±5.4	1.9 ±1.0	23.8 ±19.5	0.9 ±0.6	5.8 ±3.5	0.4 ±0.2	0.26 ±0.19
	Winter	2.0 ±0.9	2.4 ±1.7	17.9 ±13.9	0.9 ±0.6	6.0 ±4.8	0.3 ±0.2	0.28 ±0.20
	Spring	1.4 ±0.7	1.2 ±1.0	14.4 ±11.2	2.5 ±1.6	5.2 ±4.2	0.3 ±0.2	0.24 ±0.22

Table S2. Mean (±SD) elemental composition (mg/L) of different industrial wastewater, Leeuwkuil WWTP inflow and final effluents samples (n=27)

	Industry 1	Industry 2	Industry 3	Industry 4	Industry 5	WWTP Inflow	WWTP effluent
**Ag**	0.2±0.04	0.2±0.03	<MDL	0.2±0.08	0.2±0.00	0.4±0.2	2.1±0.1
**Al**	11.8±1.16	0.28±0.05	1.95±1.05	14.0±2.34	2.56±1.56	0.18±0.03	0.20±0.02
**B**	0.6±0.04	0.7±0.02	5.4±0.8	0.5±0.15	16.1±2.5	0.16±0.0	0.19±0.04
**Ca**	186±16.5	38±3.9	143±13.8	472±45.8	294±21.3	35.2±2.1	33.9±7.5
**Cr**	0.18±0.02	0.8±0.12	0.15±0.04	0.02±0.0	0.05±0.03	0.8±0.08	0.2±0.03
**Cu**	0.72±0.04	0.24±0.04	0.19±0.02	0.18±0.02	0.19±0.02	0.22±0.02	0.18±0.02
**Fe**	14.0±1.3	16.7±1.5	0.7±0.1	13.1±1.2	6.4±0.6	3.2±0.3	1.5±0.02
**K**	8.5±0.8	8.6±0.9	63.2±5.6	6.6±0.6	9.7±0.9	8.5±0.5	8.6±0.9
**Mg**	83.5±7.9	5.7±0.5	70.6±7.0	25.9±2.3	12.4±1.0	12.0±1.0	11.8±1.0
**Na**	213±18.7	432±78.6	49±4.8	97±9.0	866±85	52.8±5.0	59±6.0
**Ni**	0.3±0.01	0.26±0.02	0.2±0.01	0.4±0.12	0.2±0.0	0.24±0.04	0.18±0.02
**P**	6.2±0.04	1.1±0.02	0.8±0.04	2.6±0.06	2.3±0.04	4.5±0.01	4.0±0.04
**Pb**	4.8±0.17	0.18±0.02	0.18±0.03	0.19±0.03	0.21±0.0	0.20±0.03	0.19±0.03
**S**	3503±105	15.1±4.2	21.5±1.2	21.0±2.1	7.9±1.9	167±37.8	20±0.9
**Se**	<MDL	<MDL	<MDL	<MDL	<MDL	3.3±0.36	1.4±0.18
**Si**	11.5±8.1	6.4±0.0	0.2±0.01	2.9±0.0	0.2±0.06	4.3±0.6	1.8±0.3
**Sr**	1.8±0.4	1.8±0.6	0.8±0.08	0.4±0.1	<MDL	0.3±0.1	<MDL
**Zn**	7.41±2.04	56.7±19.03	1.47±0.74	4.07±0.21	0.26±0.05	0.20±0.01	0.16±0.03

$^{\alpha}$ MDL, minimum detectable limit of the instrument
